# Two Rare Northern *Entoloma* Species Observed in Sicily under Exceptionally Cold Weather Conditions

**DOI:** 10.1100/2012/957212

**Published:** 2012-05-03

**Authors:** Giuseppe Venturella, Alessandro Saitta, Gerlando Mandracchia, Maria Letizia Gargano

**Affiliations:** Dipartimento di Biologia Ambientale e Biodiversità, Università di Palermo, Via Archirafi 38, 90123 Palermo, Italy

## Abstract

The biology and ecology of many *Entoloma* species is still poorly known as well as their geographical distribution. In Italy, there are no studies on the influence of weather on fungal abundance and richness and our knowledge on the ecology and distribution of *Entoloma* species needs to be improved. The discovery of two *Entoloma* species in Sicily (southern Italy), reported in the literature as belonging to the habitat of north European countries, was the basis leading to the assumption that anomalous climatic conditions could stimulate the growth of northern entolomas in the southernmost Mediterranean regions. The results of this study show that the presence of northern *Entoloma* species in Sicily is not influenced by the Mediterranean type of vegetation, by edaphic or altitudinal factors but by anomalous climatic trends of precipitations and temperatures which stimulate the fructification of basidiomata in correspondence with a thermal shock during autumn.

## 1. Introduction

The taxa belonging to the genus *Entoloma* (Fr.) P. Kummer are characterized by different ecological features and requirements and only few species are considered exclusive to the Mediterranean environment.

In the wide group of *Entoloma* species, considered by mycologists as infrequent or rare, *Entoloma caeruleum* (P.D. Orton) Noordel. and *E*.* plebeioides* (Schulzer) Noordel., grows in similar ecological environments such as natural grasslands and nonmanured; intensely grazed cultivated grasslands; hayfields, open *Juniperus* heaths; dune grasslands with *Salix* spp. [1]. Additional habitats, that is, river-plain forests and coastal dune-forests, were reported for *E*.* plebeioides* [1].

In Italy, the presence of the above-mentioned entolomas is limited to few and scattered localities of northern Italy (*E*.* caeruleum*) and Sicily (*E*.* plebeioides*) [2, 3]. After the first sighting of *E*.* plebeioides* in Italy, one of the authors (G. Venturella) came to the conclusion that the presence of such northern *Entoloma* in a dry region such as Sicily was unusual and probably related to a particular climatic condition of that year [2]. Since no additional collections of *E*.* plebeioides* were subsequently available, any other hypothesis was temporarily shelved. Five years later, the finding of *E*.* caeruleum*, also never previously reported in Sicily nor in southern Italy, prompted a more in-depth investigation of the possible correlations between the growth of *Entoloma* species, usually reported from northern European countries, and the climatic trends in dry Mediterranean regions like Sicily.

## 2. Materials and Methods

In the years 2001 and 2007, the entoloma's basidiomata were collected during field excursions periodically carried out (i.e., an excursion per week in the period September–December and an excursion every 15 days from January to August) by the staff of the Laboratory of Mycology (Department of Environmental Biology and Biodiversity of the University of Palermo, Italy). The mushroom samples (2 basidiomata of *E*. *plebeioides* and 1 of *E*. *caeruleum*) were cleaned of forest debris (without washing) with a knife, transported to the laboratory in a paper box and kept at 48°C for 24 h prior to sample preparation. The identification was carried out on fresh basidiomata and the morphological characters of each of the collected specimens were evaluated with a Leica MS5 binocular microscope as follows: colours; pileus; characters of the lamellae, stipe; growth habit; type of basidiomata attachment; spore color. According to the protocol reported in Noordeloos [1], the microscopic features were evaluated with a Leica DLMB microscope using tap water and chemical reagents such as 95% ethanol, 1% Congo Red in concentrated ammonia (NH_4_OH), 10% Potassium Hydroxide (KOH), and saturated salt solution. The samples were attached to a glass microscope slide for observation (×60, ×100) and analysis. Image acquisition was done using a digital camera, incorporating a charge-coupled device (CCD) detector mounted in the optical path of the microscope. Spores sizes were based on at least 10 measurements on spores from the lamellae or stipe surface. The very essential characters of *Entoloma* species were also evaluated such as the length-width ratio of spores (Q), the presence of tetrasporic or bisporus basidia, the presence or absence of a clamp at the base of basidia, the presence of fertile lamella edge, the presence of cystidia, the type of pileipellis, and the trama structure and pigmentation. The scientific binomials of recorded taxa were referred to http://www.indexfungorum.org/Names/Names.asp. The herbarium specimens were kept in the *Herbarium Mediterraneum Panormitanum *(PAL).

## 3. Ecological Data on *Entoloma caeruleum* and *E. plebeioides* in Sicily

*E*.* caeruleum* was collected in a regional natural park, “Parco delle Madonie,” in the province of Palermo (northern Sicily). The basidiomata were found, at 600 m a.s.l., on January 10th, 2007, in a mixed wood, in the neighborhood of Collesano, a small town included in the park territory. At the site of collection, the pedological type belongs to the association “Rock outcrop-Lithic xerorthents,” evolved on limestone rocks. The soils are characterized by a basic reaction and a low exchange capacity, fully saturated in calcium [4]. The vegetation type belongs to the alliance *Erico*-*Quercion ilicis* Brullo, Di Martino and Marcenò 1977, with a tree layer represented by *Quercus suber* L., *Q*. *virgiliana* (Ten.) Ten., *Q*. *amplifolia* Guss., *Q*. *dalechampii* Ten., *Q*. *congesta* C. Presl, *Q*. *Xbivoniana* Guss., *Q*. *ilex* L., and *Fraxinus ornus* L. The shrub layer is mainly represented by *Erica arborea* L., *Arbutus unedo* L., and *Genista maderensis* Raimondo.

*E*. *plebeioides* was collected in a reforested area included in the “Parco della Favorita” (natural reserve of Monte Pellegrino) which is part of the urban centre of Palermo. The basidiomata were found, at sea level, on December 15th, 2001, in the glades of a mixed reforestation of *Pinus halepensis* Miller, *Cupressus sempervirens* L., *C*.* macrocarpa* Hartweg and *Cedrus deodara* (D. Don) G. Don. The pedological type belongs to the Mediterranean Red Soils (Typic and/or Lithic rhodoxeralfs) evolved on limestone [4]. The soils are characterized by a prevalence of sandy particles and a neutral reaction (pH 6.8–7.1). The vegetation is represented by exotic plants such as *Cedrus atlantica* (Manetti ex Endl.) Carriére, *C*. *deodara* (D. Don) G. Don f., *Cupressus arizonica* Greene, *C*. *macrocarpa* Hartw., *C*. *sempervirens* L., *Pinus halepensis* Mill., and *P*. *pinea* L.

## 4. Results and Discussion

It is well known that fungal abundance and richness depends on weather [5]. All fungi need moisture but temperature requirements can be different for different species. The majority of them need warm rather than cold temperatures, but many fungi require a drop in temperature to trigger their fructification [6]. According to Lange [7], the occurrence of fleshy fungi varies with the distribution of the precipitation throughout the season and the distribution of species within functional groups is determined by rainfall. Temperature does not appear to play a role in structuring community diversity at a regional scale [8]. Higher species diversity of macromycetes and abundance of basidiomata and ascomata are influenced by microclimatic conditions [9]. Different species, however, exhibit different fruiting periods, which vary from year to year and at different elevations and latitudes. The maximum richness of fruiting species occurs only during brief periods and differs from year to year [10]. Recent observations from Britain [11] indicate, however, that the fruiting period of macrofungi has already been affected, starting earlier in the season and lasting longer into late autumn due to climate change over the last 30 years [12, 13]. van Norman et al. [14] pointed out that in years with drought or atypical weather, fewer species produce sporocarps. The distribution and mycorrhizal efficacy of fungi forming ECM associations is also influenced by climatic and edaphic factors [13, 15]. Besides it is apparent that a wide range of variations in tolerance to edaphic and climatic factors (such as temperature extremes, drought, and soil toxicity) often occur both between and within species of mycorrhizal fungi, and these variations may represent an adaptation to specific site conditions by poorly understood genetic mechanisms [16].

The biology and ecology of many *Entoloma* species is still poorly known as well as their geographical distribution [1].

In Italy, there are no studies on the influence of weather on fungal abundance and richness, and our knowledge on the ecology and distribution of *Entoloma* species needs to be improved.

After the first finding of *E*. *plebeioides* in Sicily, previously recorded only in northern Europe, one of the authors (G. Venturella) suggested that this collection could be related to the climatic trend registered in the city of Palermo during the year 2001 [3]. *E*.* caeruleum* was collected five years later, under the same climatic conditions of the year 2001, in a different locality one hundred kilometres away from Palermo. *E*.* caeruleum*, widely recorded in northern Europe, and never previously reported in Sicily, and up until then only collected in two localities of northern Italy [17] subjected to a very different climate.

**Figure 1:**
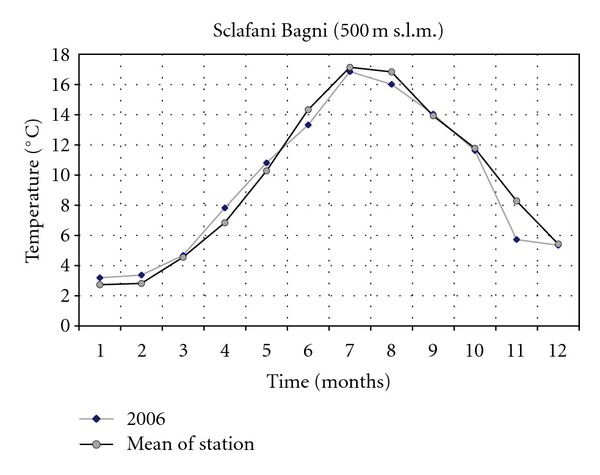
Daily minimum temperature trend in 2006 compared with the mean trend registered in the thermopluviometric station.

**Figure 2:**
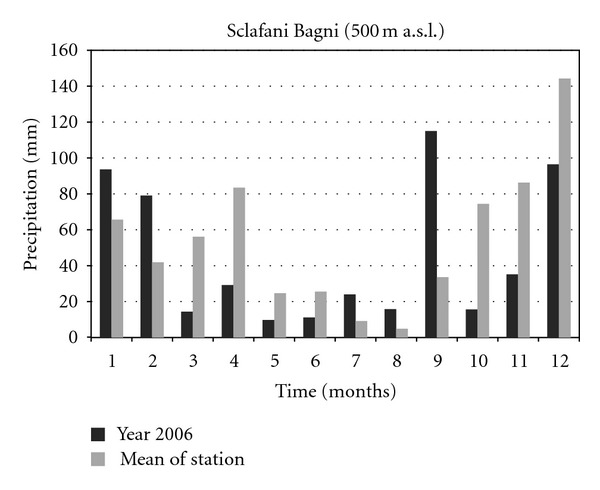
Monthly rainfall trend in 2006 compared with the mean trend registered in the thermopluviometric station.

**Figure 3:**
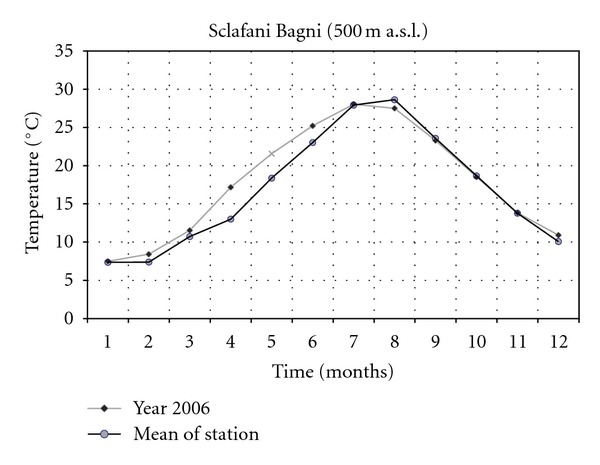
Soil mean temperature trend in 2006 compared with the mean trend registered in the thermopluviometric station.

**Figure 4:**
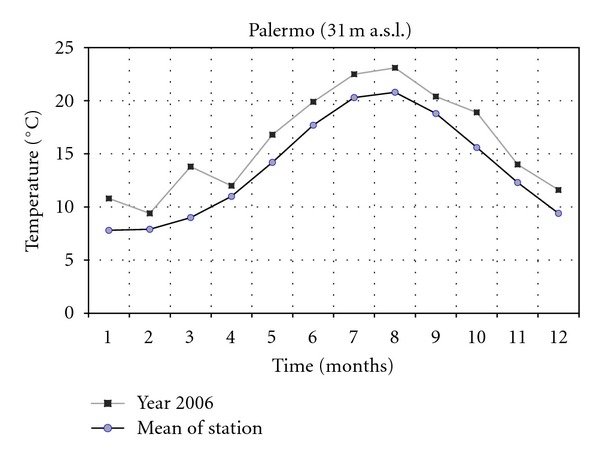
Daily minimum temperature trend in 2001 compared with the mean trend registered in the thermopluviometric station.

**Figure 5:**
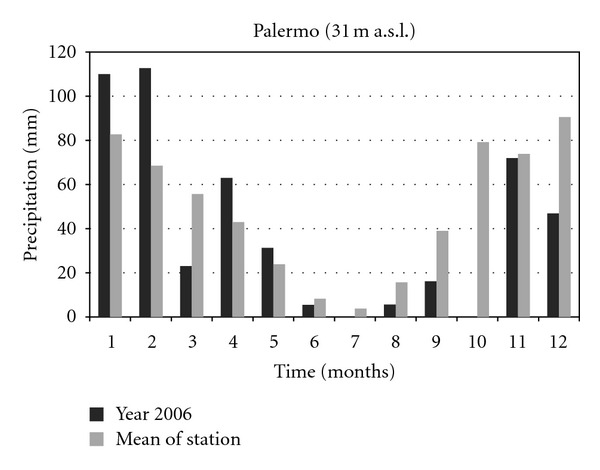
Monthly rainfall trend in 2001 compared with the mean trend registered in the thermopluviometric station.

The climatic data concerning the years 2001 and 2006 of two thermopluviometric stations of the province of Palermo, that is, the Astronomical Observatory of Palermo (72 m a.s.l.) and the station of Sclafani Bagni (500 m a.s.l.) were compared. In the year 2006 in Sclafani Bagni, the thermopluviometric station closest to the collection site of *E*.* caeruleum*, the temperatures of the quarter October-November were characterized by a considerable cooling down of the mean monthly of daily minimum temperatures (Figure 1). The minimum temperature of November was lower than 2.6°C (31% less than the medium value of the station corresponding to 8.3°C). The rainfall of September (Figure 2) exceeded the medium values by 242% (115.0 mm in comparison with the medium value of 33.7 mm). In October and November 2006, a reduction of 79% and 59% of rainfall was, respectively, registered (15.6 mm in comparison with the medium value of 74.5 mm and 35.2 mm in comparison with the medium value of 86.3 mm). As a consequence, an increase of the daily thermic excursions was registered and the climate of the *E*.* caeruleum* collection-site increased its aspect of continentality. Besides, in the station of Sclafani Bagni, the daily mean temperatures of soil (Figure 3) from March to July resulted higher than the medium values of the station. The dry period was extended from March to June, while July and August were more rainy in comparison to the mean values of rainfalls with precipitations of lower values but double the medium values of the station. In 2001, the data available from the Astronomical Observatory of Palermo, the thermopluviometric station closest to the collection site of *E*.* plebeioides*, showed remarkable analogies with data recorded in 2006 in the thermopluviometric station of Sclafani Bagni. In the town of Palermo, in October 2001, the autumnal climatic trend was characterized by high temperatures, sometimes higher than 5°C in comparison with the mean values of the station (Figure 4). On the other hand, rainfalls were extremely rare. The regional mean deviation of rainfall values in 2001 compared with the thirty-year medium value of rainfall (corresponding to ca. 78 mm) showed a reduction of 97% in the precipitation values (Figure 5). The precipitations slightly increased only in November 2001. Besides, in autumn the daily mean temperatures were lower than the medium values usually registered by the Astronomical Observatory of Palermo. Data on soil daily mean temperatures were not available from the thermopluviometric station of Palermo.

According to the thermopluviometric data, a link between climatic trend and the growth of entolomas in the Mediterranean environments can be identified. The results of this study show that the presence of northern *Entoloma* species in Sicily is not influenced by the Mediterranean type of vegetation, by edaphic, or by altitudinal factors but by anomalous climatic trends of precipitations and temperatures which stimulate the fructification of basidiomata in correspondence with a thermal shock during autumn.
